# Causal effect of vitamin D on myasthenia gravis: a two-sample Mendelian randomization study

**DOI:** 10.3389/fnut.2023.1171830

**Published:** 2023-07-19

**Authors:** Yidan Fan, Huaiying Huang, Xiangda Chen, Yuexuan Chen, Xiashi Zeng, Fenwei Lin, Xu Chen

**Affiliations:** ^1^The First Clinical Medical College, Guangzhou University of Chinese Medicine, Guangzhou, China; ^2^Guangdong East Hospital of Guangmei Development Zone Hospital of the Third Affiliated Hospital of Sun Yat-sen University, Meizhou, China; ^3^The First Affiliated Hospital of Guangzhou University of Chinese Medicine, Guangzhou, China

**Keywords:** myasthenia gravis, vitamin D, Mendelian randomization, genetics, causality

## Abstract

**Introduction:**

Observational studies suggest that vitamin D supplementation may be effective in preventing myasthenia gravis (MG). However, the causal relationship between circulating vitamin D levels and MG remains unclear. This study aimed to examine the genetic causality of circulating vitamin D and MG using data from large population-based genome-wide association studies (GWAS).

**Methods:**

SNPs (single nucleotide polymorphisms) strongly associated with exposure were selected. Two-sample Mendelian Randomization (MR) was performed with inverse variance weighting (IVW), MR-Egger (Mendelian randomization-Egger), weight median and MR-PRESSO (Mendelian randomization pleiotropy residual sum and outlier) methods. Heterogeneity was tested via IVW and MR-Egger. Pleiotropy was tested using MR-Egger intercept test and MR-PRESSO method. MR-PRESSO was also used to detect outliers. Leave-one-out analysis was used to identify SNPs with potential effect. Reverse MR analysis was also performed.

**Result:**

In IVW, circulating vitamin D levels had no causal effect on MG [OR = 0.91 (0.67–1.22), *p* = 0.532] and MG had no causal effect on circulating vitamin D [OR = 1.01 (099–1.02), *p* = 0.663]. No heterogeneity or pleiotropy was observed (*p* > 0.05). Other MR methods also agreed with IVW results.

**Conclusion:**

This study provides the causal relationship between genetically predicted circulating vitamin D levels and MG and provides new insights into the genetics of MG.

## Introduction

Myasthenia gravis (MG) is an autoimmune disease characterized by a disorder of transmission of neuromuscular junctions mediated by autoantibodies. In most cases, pathogenic antibodies are directed against acetylcholine receptors (AChRs), which are the most common type of antibodies. Additionally, antibodies against other components of the postsynaptic membrane, such as muscle-specific receptor tyrosine kinase (MuSK), low-density lipoprotein receptor-related protein 4 (LRP4), and ryanodine receptor (RyR), are being developed (1). The prevalence of MG is estimated to be between 150 and 250 per million worldwide, and the annual incidence is estimated to be between 4 and 10 per million (2). There is a bimodal age range for the onset of MG with the peak being at the age of 30 years and a steady increase in incidence as one ages beyond 50 (3). Genome-wide association studies have been conducted to examine genetic risk factors for MG, a disease that is mediated by genetic and environmental factors.

As an antibody-mediated autoimmune disease, MG is dependent on T cells (4). Researchers have found that Treg cells, a subpopulation of T cells which suppresses the activation of other immune cells, are mainly responsible for suppressing T-cell activation (5). Previous studies have revealed that the number of Treg cells in peripheral blood lymphocytes of MG patients is reduced when compared to normal subjects (6). On the other hand, vitamin D3 appears to have a regulatory effect on Treg cells in patients suffering from MG (7). According to previous cohort and cross-sectional studies (8, 9), patients with MG have lower vitamin D levels than those in the general population. In patients with MG, vitamin D supplementation has been found to improve fatigue scores (10–12). It was noted, however, that a randomized controlled trial found no clinically significant difference between patients taking vitamin D and those taking a placebo (13).

Thus, vitamin D deficiency is considered to be a potential risk factor for MG. Despite this, no causal link has been established between serum vitamin D levels and MG risk. A randomized controlled trial is extremely difficult to conduct, as it requires a lot of manpower and material resources. Sometimes, ethical considerations prevent research on a particular factor from being conducted. Additionally, there may be some confounding factors in traditional studies that are difficult to eliminate, and there may be a causal inversion between exposure and outcome.

As an alternative to conventional methods, Mendelian randomization (MR) employs genetic variation as an exposure indicator for testing exposure’s causal effect on outcome (14), thus overcoming their limitations. During the course of this study, we pooled statistics from two large genome-wide association studies (GWAS) conducted on vitamin D circulating levels and MG in order to determine whether circulating vitamin D levels are causally related to MG.

## Materials and methods

### Study design

**Figure 1:**
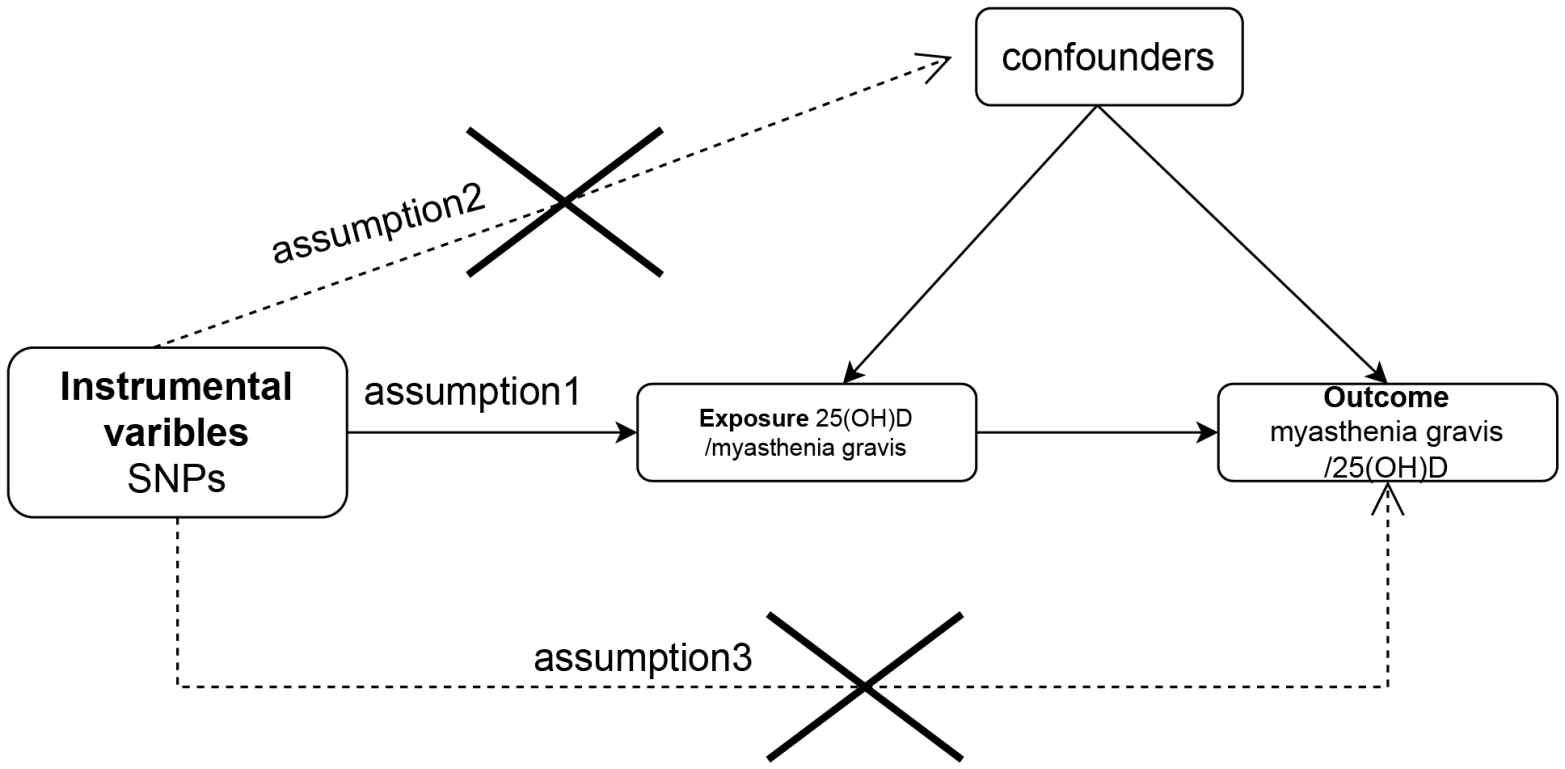
Flow chart of the study design for the Mendelian randomization study. The Mendelian randomization approach is based on three assumptions: (1) instrumental variables are closely related to exposure, (2) instrumental variables are independent of any confounding factors, and (3) instrumental variables affect outcomes only by way of exposure and not by other means. SNP, single-nucleotide polymorphism.

**Figure 2:**
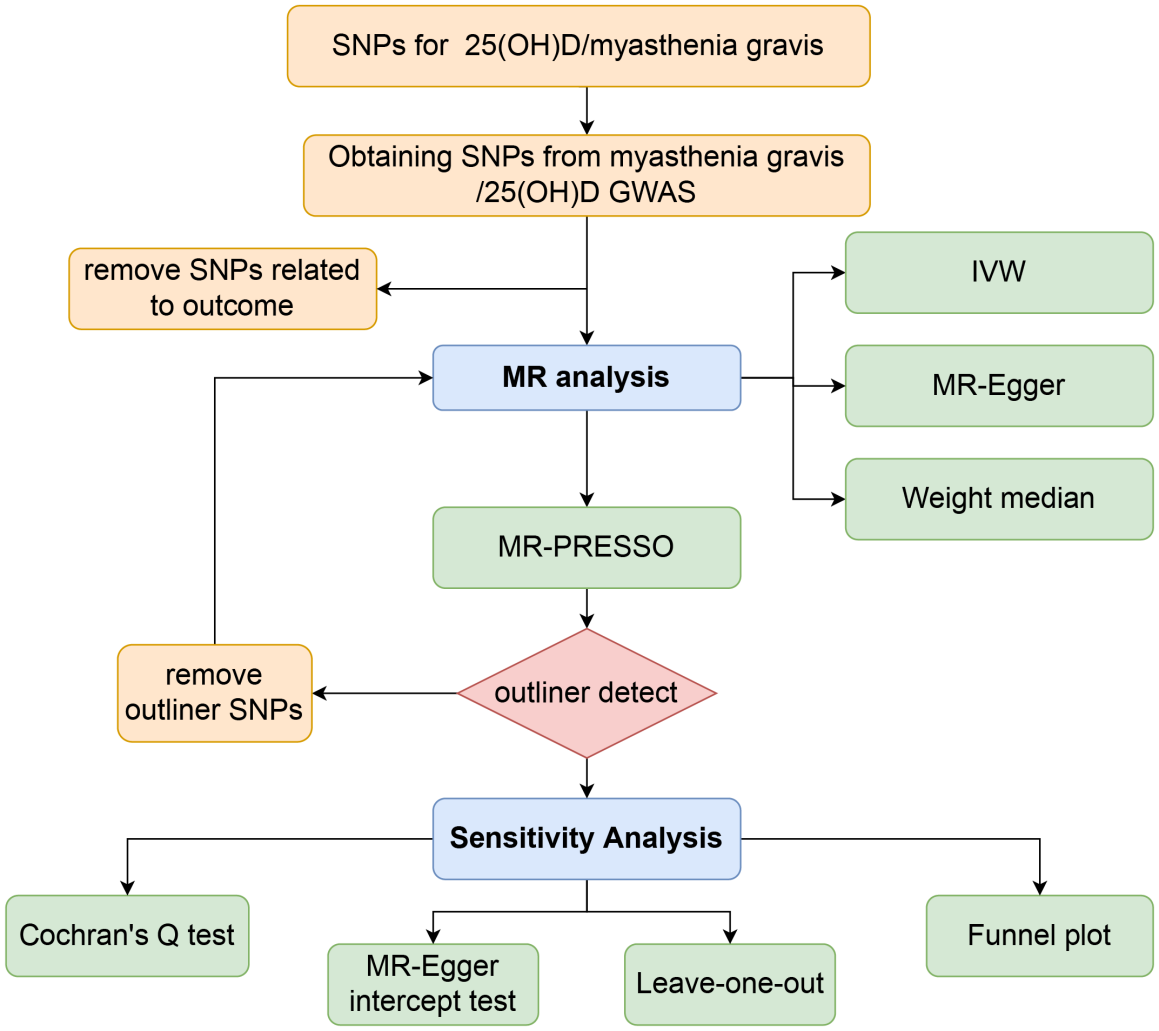
Workflow of Mendelian randomization study revealing causality between 25(OH)D and risk of myasthenia gravis. SNP, single-nucleotide polymorphism; IVW, inverse variance weighted; MR, Mendelian randomization; MR-PRESSO, MR pleiotropy residual sum and outlier.

There were three components to the Mendelian randomization study design: (1) identifying genetic variants that could be used as instrumental variables for vitamin D, (2) obtaining data summaries from genome-wide association studies (GWAS) for the purpose of genetic instrumentation, and (3) obtaining and harmonizing summary data for single nucleotide polymorphism (SNP) results that were utilized for determining GWAS genetic instruments’ effect on myasthenia gravis risk (Figure 1). Similar steps were taken in reverse Mendelian randomization. The flowchart of the study is illustrated in Figure 2.

### GWAS summary data for vitamin D

In GWAS for serum 25(OH)D, data were exacted from a meta-analysis of vitamin D GWAS undertaken by the UK Biobank study and the Sunshine consortium, involving 495,613 European ancestry participants (15). A data set of 417,580 European individuals (age range 40 to 69 years) with European ancestry was retrieved from the UK Biobank (UKB) with the aim of investigating the association between SNPs and serum 25(OH)D concentrations, as well as phenotypic, genotypic, and clinical information. It was performed a linear mixed model GWAS to identify associations between genetic variants and 25(OH)D levels, followed by a meta-analysis using the results from the SUNLIGHT consortium GWAS (sample size of 79,366), which included 2,579,297 SNPs (16). A detailed report on quality control and statistical analysis has already been published (15). At the genome-wide significance level (*p* < 5 × 10^−8^), 118 independent SNPs were identified as being associated with 25(OH)D levels (r2 threshold <0.001, 10,000 kb). There were no overlaps or linkage disequilibriums with known risk loci for myasthenia gravis (r2 < 0.001). According to the results of this study, 118 SNPs explained 41.4% of the variance in total 25(OH)D. Table 1 summarises the GWAS studies and datasets employed in the study.

**Table 1:** Characteristics of data sources used in the Mendelian randomization study.

Traits	Data sources	Sample size	Ancestry
25(OH)D	UK Biobank and SUNLIGHT consortium	495,613	European
MG	Chia et al. (17)	38,423	European

MG, myasthenia gravis.

### GWAS summary data for myasthenia gravis

Meta-GWAS data for MG were collected from the largest meta-GWAS conducted in the United States and Italy (1,873 patients and 36,370 age-and sex-matched controls) (17). The study enrolled only patients with antiacetylcholine receptor antibody-positive (AChR+) MG, and patients with positive muscle-specific kinase antibody (MuSK+) test results were excluded from participation. Institutional review boards at all participating institutions approved the study, including Johns Hopkins University, the National Institute on Aging (protocol 03-AG-N329), the University of Pisa, and the Catholic University of Rome.

### Validation of instrumental variable validity and correlation for vitamin D

In order to determine the effectiveness of the selected instrumental variable SNP of vitamin D, we use Phenoscanner (18) to further obtain the gene region corresponding to the SNP. At the same time, in order to further clarify the function of the gene region corresponding to the vitamin D instrumental variable, we used the ClusterProfiler R package to evaluate the Gene Ontology (GO) (19) and Kyoto Encyclopedia of Genes and Genomes (KEGG) pathway enrichment analysis (20). A value of *p* of 0.05 was considered statistically significant. The R packages enrichment plot and ggplot2 (21) were used to visualize the data. To further visualize the association of SNPs with phenotypes, we performed a Manhattan plot visualization using the qqman package (22).

### Statistical analysis

#### Mendelian randomization analysis

We assessed the strength of interpretation of the quantitative instrumental variables by calculating the F statistic as F = *R*^2^ × (N − 2)/ (1 − *R*^2^). *R*^2^ is the proportion of serum vitamin D levels explained by each instrumental variable and N is the sample size of the GWAS associated with serum vitamin D (23). R2 was calculated as (2xEAFx(1−EAF) × beta^2^)/[(2xEAFx(1−EAF) × beta^2^) + (2xEAFx(1−EAF) × N × SE (beta)^2^)]. It is important to note that EAF stands for effect allele frequency, beta represents gene effect estimated on serum vitamin D and SE (beta) stands for standard error of gene effect estimation (beta) (24).

To assess the potential causal relationship between circulating 25(OH)D levels and the risk of MG, we used the inverse variance weighting (IVW) method as the primary analysis. For the purpose of estimating the causal effects of multiple monogenic IVs (25), the IVW method incorporates the Wald ratio method, which yields unbiased results if horizontal pleiotropy is not present. Based on the IVW method, effect sizes are calculated based on weighted regression of regression coefficients, without accounting for intercepts (26). As well as weighted median method, MR-Egger method was also utilized for the analysis of Mendelian randomization. Once the weight of the causal effect calculated by the effective instrumental variants reaches 50%, a consistent estimate of the causal effect can be obtained using the weighted median method (27). Also, horizontal pleiotropic outliers were detected and corrected using the Mendelian randomized pleiotropic residuals and outliers (MR-PRESSO) method (28). To assess global heterogeneity, a regression analysis was conducted, regressing SNP-outcome associations on SNP-exposure associations, and comparing observed and expected SNP distances.

To test for any inverse association, we also conducted a bidirectional Mendelian randomization analysis using vitamin D as the outcome and MG as the exposure.

### Sensitivity analysis

Further sensitivity analysis was performed using Cochran’s Q statistic, funnel pot, and leave-one-out tests, as well as the MR-Egger intercept test. Heterogeneity of instrumental variables was indicated when the value of *p* of Cochran’s Q test was less than 0.05. The validity of the genetically predicted relation between circulating 25(OH)D and MG risk was further tested using the “leave one out” method. Reestimating the effects of the remaining SNPs was performed by removing each SNP separately and applying the IVW method. Fluctuations in the results before and after SNP deletion indicate the stability of the causal relationship between exposure variables and outcome. In MR-Egger’s diagram, the intercept term represents the mean pleiotropic effect of instrumental variables. The presence of directional pleiotropy (29) is indicated by a non-zero intercept. As a final step, phenoscanner^1^ was employed to query other relevant features in previously published GWAS (18) that might influence the results. SNPs associated with other genome-wide significant traits will be excluded from the IVs, after which MR analysis will be performed again using the remaining SNPs. MR analysis was conducted using R (version 4.1.2), in conjunction with the packages “Mendelian Randomization” and “MR-presso.” A value of *p* < 0.05 was considered statistically significant.

## Result

### Characteristics of instrumental variables

As presented in Supplementary Tables S1, S2, the instrumental variables are presented in detail along with their estimated effect estimates on 25(OH)D and MG, respectively. Based on the GWAS data, 118 instrumental variables were identified as statistically significantly associated with 25(OH)D (*p* < 5 × 10^−8^) after removing chain imbalance (r2 < 0.001, 10,000 kb). A total of nine SNPs were missing from the pooled GWAS data for MG, and rs1558902 with an intermediate allele frequency was also removed. A total of 108 SNPs were eventually included for further analysis (Table 2 and Supplementary Table S1). All of the F-statistics for the instrumental variables of 25(OH)D exceeded 10, which indicates that the instrumental variables are unlikely to be weakly biased.

**Table 2:** Mendelian randomization of circulating 25(OH)D levels and the risk of myasthenia gravis.

Exposure	Outcome	MR method	SNP (*n*)	OR(95%CI)	*p*
25(OH)D	MG	IVW (random effects)	108	0.91 (0.67,1.23)	0.535
		IVW (fixed effects)	108	0.91 (0.67,1.23)	0.532
		MR Egger	108	0.72 (0.46,1.15)	0.171
		Weighted median	108	0.89 (0.58,1.37)	0.599
		MR-PRESSO	108	0.93 (0.79,1.08)	0.623

MG, myasthenia gravis; IVW, inverse variance weighted; MR-Egger, Mendelian randomization-Egger; MR-PRESSO, Mendelian randomization pleiotropy residual sum and outlier.

In the reverse MR analysis, five significantly correlated (*p* < 5 × 10^−8^) SNPs were used in the subsequent reverse MR analysis after removal of linkage disequilibrium, respectively, and there was no evidence that these SNPs were subject to weak IV bias (F statistic greater than 10) (Table 1 and Supplementary Table S2).

### Correlation between genetically predicted 25(OH)D levels and the risk of myasthenia gravis

As shown in Table 2, in the primary analysis, the IVW approach did not demonstrate a causal relationship between genetically predicted levels of 25(OH)D and the risk of MG (OR = 0.90; 95%CI, 0.67–1.22, *p* = 0.532). The results of the MR-Egger, weighted median, and MR PRESSO analyses were consistent with those of the IVW analysis (Table 2), indicating the reliability of the MR analysis results. Furthermore, the MR-PRESSO method did not reveal any outliers.

### Validation of instrumental variable validity and correlation for vitamin D

As shown in Supplementary Figure S3, the functional enrichment results for both genes associated with high vitamin D levels and genes associated with low vitamin D levels were highly focused on cholesterol metabolism. As shown in Supplementary Figure S4 (SNPs included in the study as vitamin D are marked with green dots), the instrumental variables included in our Mendelian randomization analysis as circulating vitamin D levels were all highly correlated with circulating vitamin D levels (i.e., greater than the genome-wide significance of 5 × 10^−8^ indicated by the red line in the figure).

### Correlation of genetically predicting risk of myasthenia gravis with 25(OH)D levels

Inverse MR results are presented in Table 3. According to the IVW method with a fixed-effects model, there was no causal relationship between MG and serum 25(OH)D levels [OR = 1.01; 95% CI, (0.99–1.02), *p* = 0.093]. Additionally, the MR-PRESSO method, the MR-Egger method, and the IVW method for the random effects model (Table 3) all produced results that confirmed the IVW method for the fixed effects model, illustrating the reliability of the MR inverse analysis. In the MR-PRESSO method, an outlier rs4409785 was detected, and heterogeneity disappeared after removal of the outlier, leaving the fixed effects model IVW results unchanged [OR = 1.00, 95% CI, (0.99–1.01), *p* = 0.66]. According to the IVW method of random effects model, the weight median method, MR-PRESSO method, and MR-egger method, MG and serum 25(OH)D levels do not appear to be causally related.

**Table 3:** Mendelian randomization of the risk of myasthenia gravis and circulating 25(OH)D levels.

Exposure	Outcome	MR method	SNP (*n*)	OR(95%CI)	*p*
MG	25(OH)D	IVW (random effects)	5	1.01 (0.99,1.02)	0.379
		IVW (fixed effects)	5	1.01 (0.99,1.01)	0.093
		MR Egger	5	0.96 (0.88,1.05)	0.462
		Weighted median	5	1.01 (0.99,1.02)	0.382
		MR-PRESSO	5	1.01 (0.99,1.01)	0.429
		IVW (random effects) (Outliner removed)	4	1.00 (0.99,1.01)	0.741
		IVW (fixed effects) (Outliner removed)	4	1.00 (0.99,1.01)	0.663
		MR Egger (Outliner removed)	4	0.98 (0.91,1.05)	0.561
		Weighted median (Outliner removed)	4	1.00 (0.99,1.01)	0.748
		MR-PRESSO (Outliner removed)	4	1.00 (0.99,1.01)	0.624

MG, myasthenia gravis; IVW, inverse variance weighted; MR-Egger, Mendelian randomization-Egger; MR-PRESSO, Mendelian randomization pleiotropy residual sum and outlier; SNP, Single nucleotide polymorphism.

### Sensitivity analysis

According to the MR-Egger intercept test and the MR-PRESSO test (Table 4), neither instrumental variables of 25(OH)D nor instrumental variables of MG showed horizontal pleiotropy (*p* > 0.05). It was found, however, that in the Cochran’s Q statistic for the instrumental variable of MG, the *p* value was less than 0.05, suggesting heterogeneity between the SNPs. It was found that this heterogeneity was no longer observed following the removal of the outlier rs4409785 identified by the MR-PRESSO method. The impact of individual SNPs on the overall causal correlation effect was not significant (Supplementary Figures S1C, S2C). Forest plots and funnel plots are shown in Supplementary Figures S1(B,D), S2(B,D). In all funnel plots, there is no indication of heterogeneity since the plots are symmetrical.

**Table 4:** Sensitivity analysis of the causal association between 25(OH)D and the risk of myasthenia gravis.

Exposure	Outcome	SNP selection	SNP (*n*)	Cochran Q test		MR-Egger	
				Q value	*p*	Intercept	*p*
25(OH)D	MG	Complete	108	108.67	0.436	0.0084	0.199
MG	25(OH)D	Complete	5	14.62	0.005	0.0131	0.394
MG	25(OH)D	Outliner removed	4	5.19	0.158	0.0077	0.531

MG, myasthenia gravis; SNP, single nucleotide polymorphism; MR-Egger, Mendelian randomization-Egger.

## Discussion

As far as we know, this is the first large-scale MR analysis that examines the causal relationship between genetically predicted circulating vitamin D levels and the risk of developing MG. Based on the largest recently published GWAS of MG for Mendelian randomization analysis, we found no evidence that circulating vitamin D impacts the risk of developing MG.

In our study, we found that the functional enrichment results of both genes related to high vitamin D levels and genes related to low vitamin D levels were highly concentrated in cholesterol metabolism. Vitamin D is one of the important products of cholesterol metabolism. Cholesterol in human skin is converted to 7-dehydrocholesterol through dehydroxylation. UV-radiation regulates the transition of 7-dehydrocholesterol to pre-vitamin D3, which then isomerises to form cholecalciferol (30). Cholecalciferol transforms to calcidiol (25(OH)D), which subsequently transforms to the active form calcitriol (1,25(OH)2D) (31, 32). Therefore, we believe that the instrumental variable SNP used in our Mendelian randomization analysis is highly correlated with circulating vitamin D levels and can be used as an instrumental variable for circulating vitamin D levels.

According to previous studies, vitamin D may have immunomodulatory properties, which increases tolerance to autoantigens (33). A recent meta-analysis suggests that serum vitamin D levels are significantly lower in MG patients than in healthy controls (34). It is therefore possible that vitamin D deficiency may play a role in the development of autoimmune diseases like MG. It ought to be noted, however, that previous studies have had different biases. For example, patients with hypercalcemia due to immobilization may also have reduced vitamin D levels (35) while patients with MG may receive plasma exchange, which may deplete serum levels of vitamin D (36). A patient with MG treated with high doses of vitamin D showed a positive prognosis in two previous case reports (37, 38). It has been demonstrated in a study by Askmark et al. (10) that vitamin D supplementation improved fatigue in patients with muscle weakness due to MG. However, Okparasta et al. (13) failed to demonstrate a significant improvement in the severe muscle weakness composite score (MGCS) following vitamin D supplementation.

Therefore, it is difficult for previous studies to determine whether vitamin D deficiency is a real risk factor or merely a consequence of reduced mobility. Mendelian randomization (MR) is an effective method for overcoming biases by utilizing genetic variation as an indicator of exposure to determine whether exposure impacts outcome (14).

There are some advantages to our study. First, the MR design was based on three main hypotheses, each of which was demonstrated using a different method. The four MR methods did not show a causal relationship between 25OHD levels and the risk of MG. In addition, their results were robust, with no significant bias associated with their respective methods. Secondly, the use of two-sample Mendelian randomization enabled us to conduct the largest genome-wide association study on MG yet undertaken, improving the likelihood of establishing a causal relationship between 25OHD levels and MG risk. There was less likelihood of confounding and reverse causality bias in this study than in previous routine observational studies. Last but not least, we performed a reverse MR analysis to rule out the presence of reverse causality.

A few limitations should also be noted regarding our study. First, the study was conducted among participants of European ancestry, so the results cannot easily be generalized to other ethnic groups with different lifestyles and cultural backgrounds. Secondly, due to the fact that Mendelian randomization employs random assignment of genetic variation in order to assess the causal hypothesis of extrapolation, it is difficult to fully differentiate between mediation and pleiotropy using MR. Further, the majority of the MG patients involved in this study were AchR-positive, making it more difficult for the results of this study to be extrapolated to all patients with MG. Nonetheless, AchR-positive MG patients accounted for 85% of all MG patients (39), so we believe our findings are generally applicable. In addition, the current MG-related GWAS data do not yet allow further typing according to the affected muscle groups of patients, which further limits the refinement of the findings of this study. In future studies, more GWAS studies for patients with different antibody types of MG such as MuSK, LRP4 or even antibody-negative are needed, as well as typing according to the site of myoplasmic involvement of patients. Lastly, although our MR study may provide the strongest evidence for a causal relationship between genetically predicted vitamin D levels and risk of MG, there was no examination of vitamin D’s influence on disease activity in patients with established MG.

## Conclusion

Under mendelian randomization assumptions, our study suggested that no causal relationship was found between vitamin D deficiency and myasthenia gravis, neither did vitamin D deficiency pose a risk factor for the development of the disease. In the future, there is a need for a larger sample size and GWAS data of other types of myasthenia gravis patients to update the conclusion.

## Data availability statement

The datasets presented in this study can be found in online repositories. The names of the repository/repositories and accession number(s) can be found at: GWAS summary data for myasthenia gravis are available for download from the GWAS Catalog database (http://ftp.ebi.ac.uk/pub/databases/gwas/summary_statistics/GCST90093001-GCST90094000/GCST90093061/), and GWAS meta-analysis data for vitamin D are available at https://www.cnsgenomics.com/content/data.

## Ethics statement

Ethical review and approval was not required for the study on human participants in accordance with the local legislation and institutional requirements. Written informed consent for participation was not required for this study in accordance with the national legislation and the institutional requirements.

## Author contributions

XuC conceived the study. FL obtained the data from public databases. YC, YF, and XZ performed the Mendelian randomization analysis as well as the integration of the results. HH and XiC made contributions to the revision of the manuscript, especially the supplementary material and the writing of the manuscript part. All authors contributed to the article and approved the submitted version.

## Funding

This work was supported by the National Natural Science Foundation of China of Youth Program (No. 81904145).

## Conflict of interest

The authors declare that the research was conducted in the absence of any commercial or financial relationships that could be construed as a potential conflict of interest.

## Publisher’s note

All claims expressed in this article are solely those of the authors and do not necessarily represent those of their affiliated organizations, or those of the publisher, the editors and the reviewers. Any product that may be evaluated in this article, or claim that may be made by its manufacturer, is not guaranteed or endorsed by the publisher.

## Footnotes

^1^ http://www.phenoscanner.medschl.cam.ac.uk/
