# Supplementary figures and images for: Causal effect of vitamin D on myasthenia gravis: a two-sample Mendelian randomization study
Source: Front Nutr. 2023 Jul 19;10:1171830. doi: 10.3389/fnut.2023.1171830 (PMC10394469; doi:10.3389/fnut.2023.1171830)

A

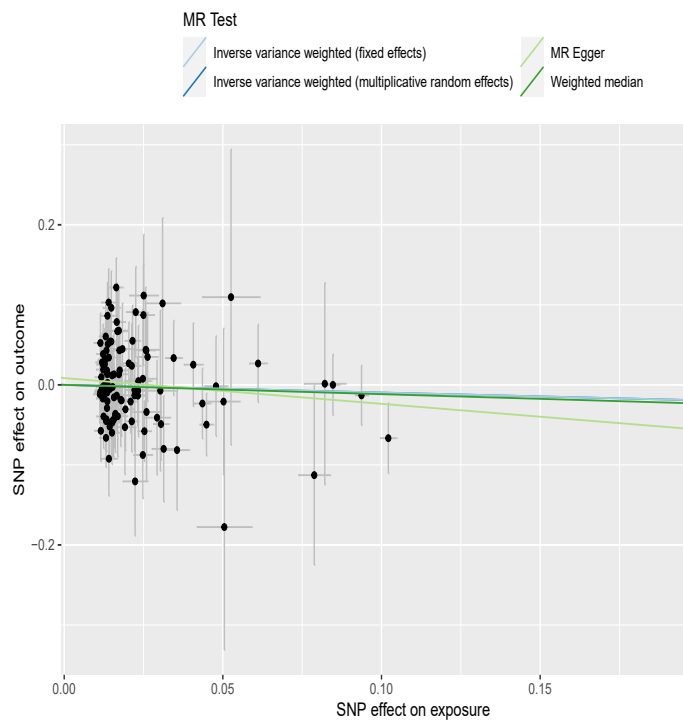

B

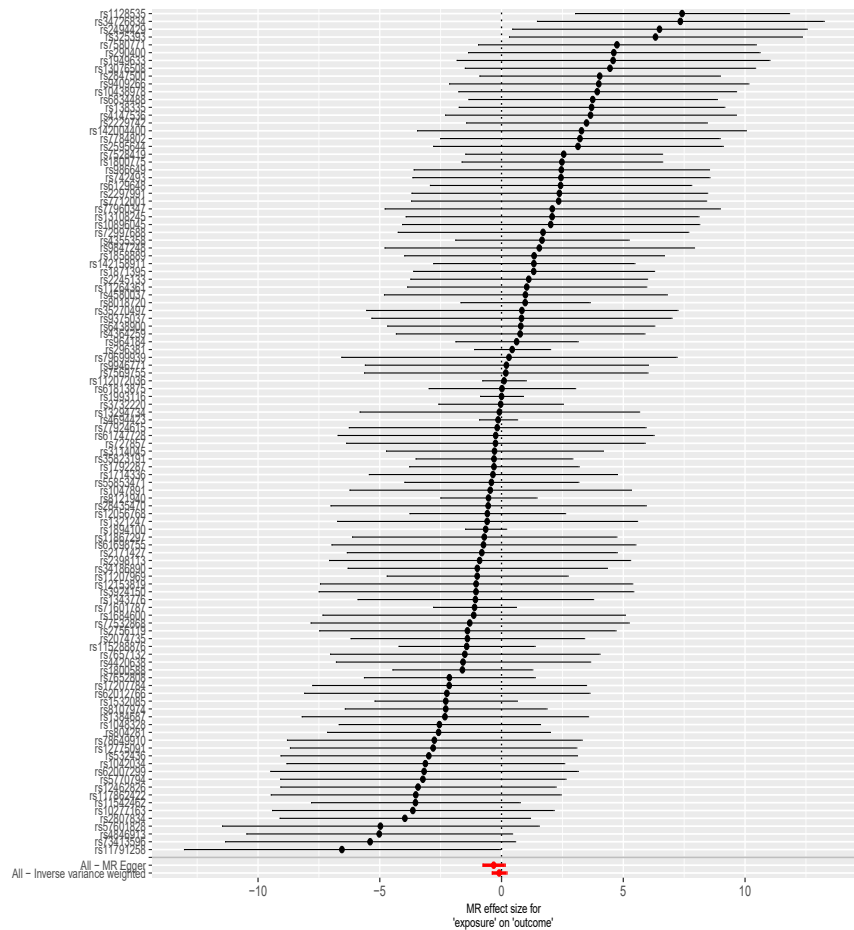

C

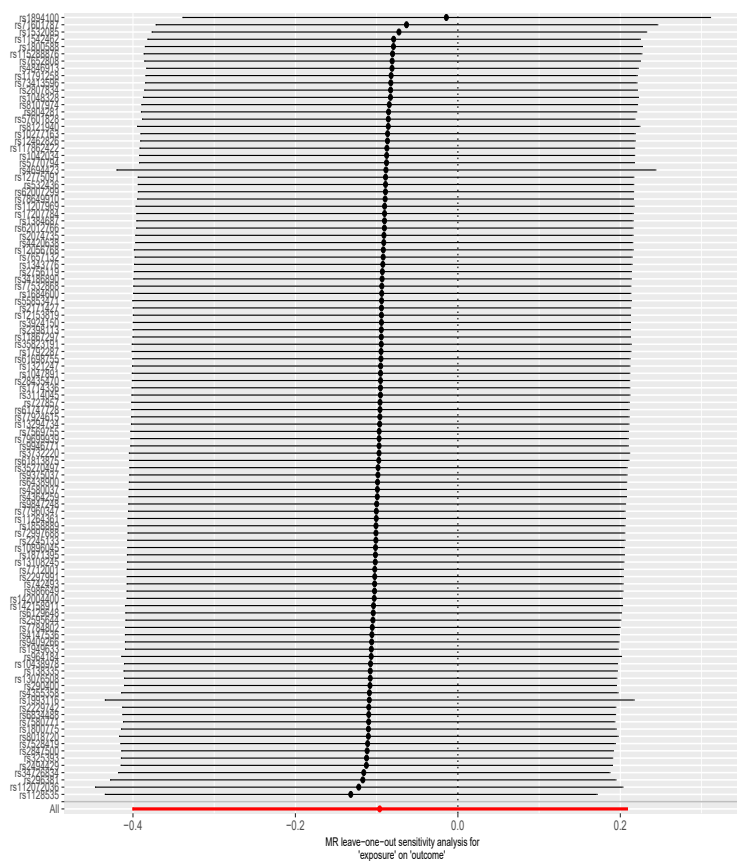

D

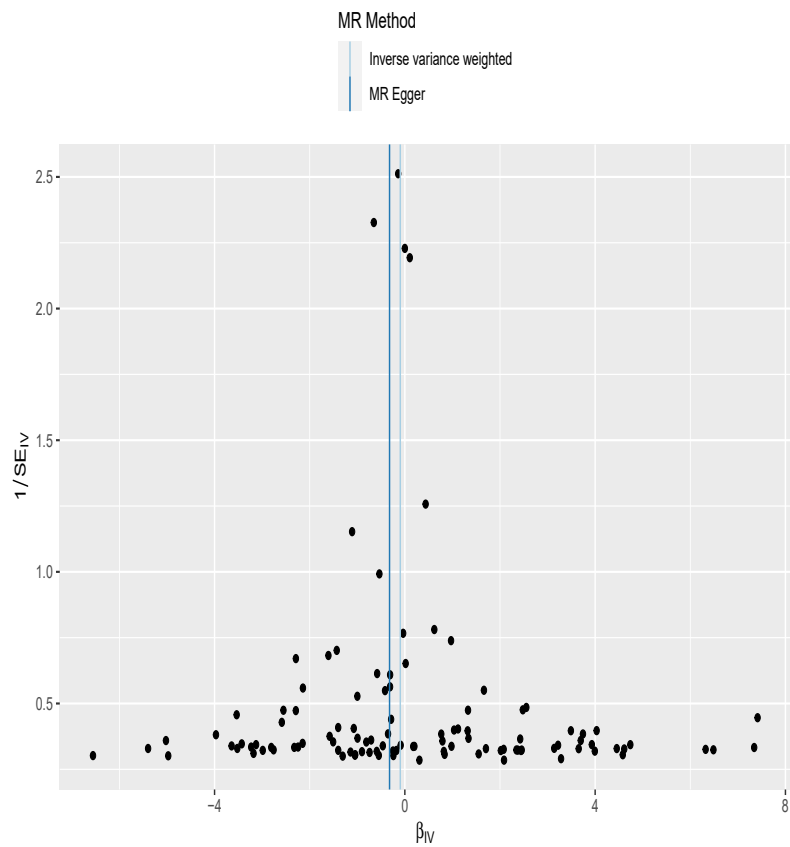

Supplement: Supplementary file 1 [file Image_1.PDF]

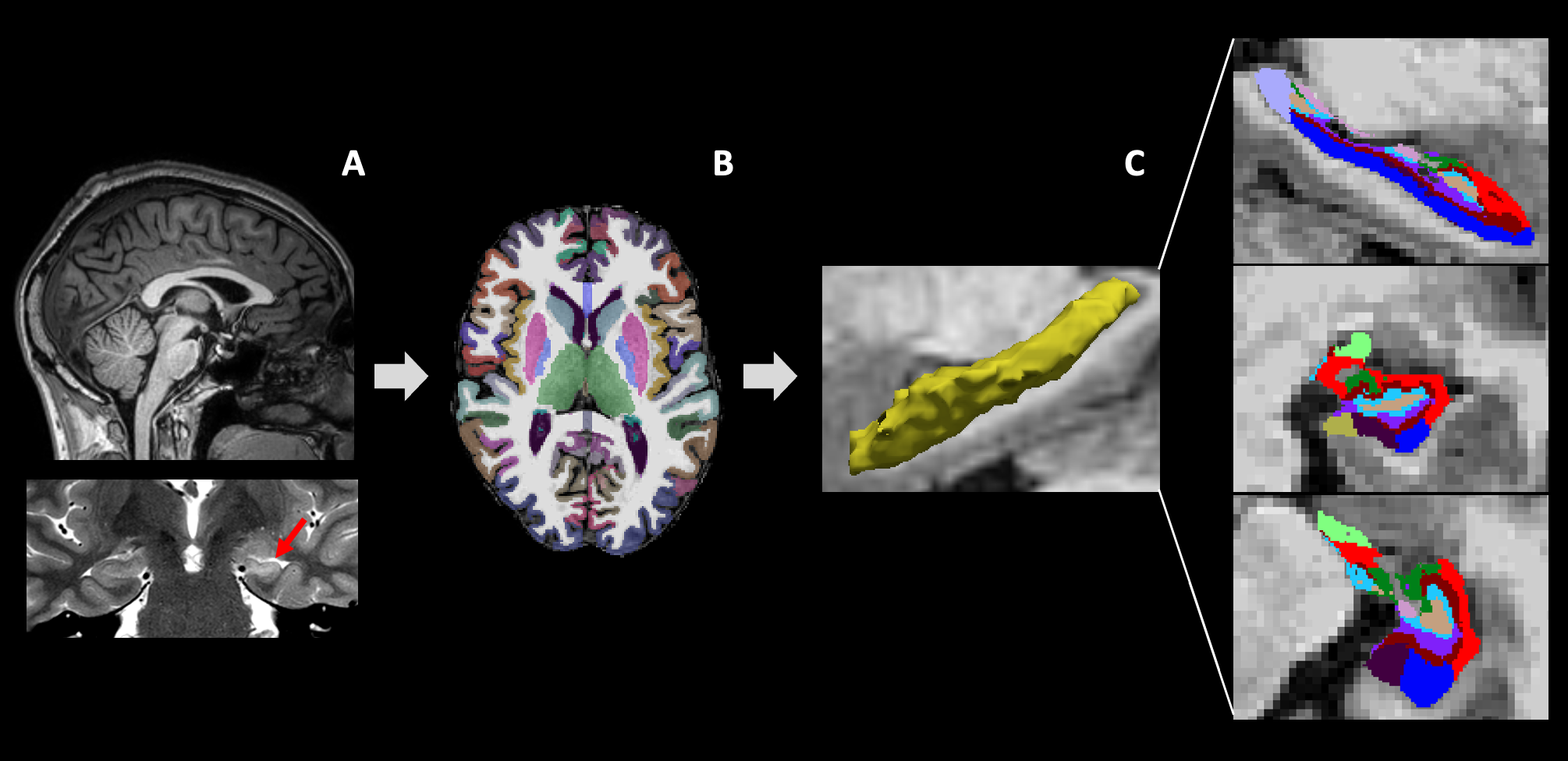

Supplement: Supplementary file 2 [file Image_1.png]

A

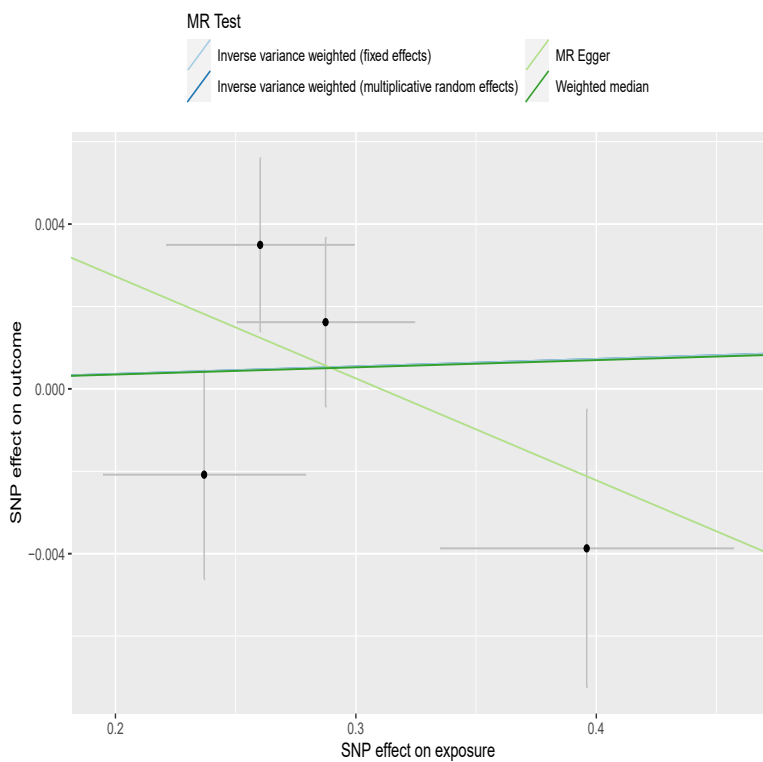

B

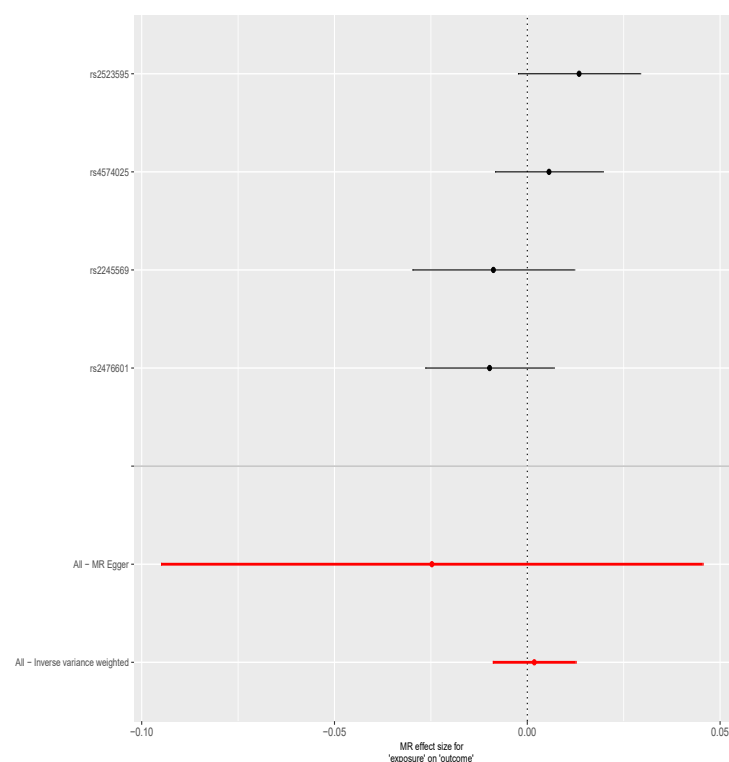

C

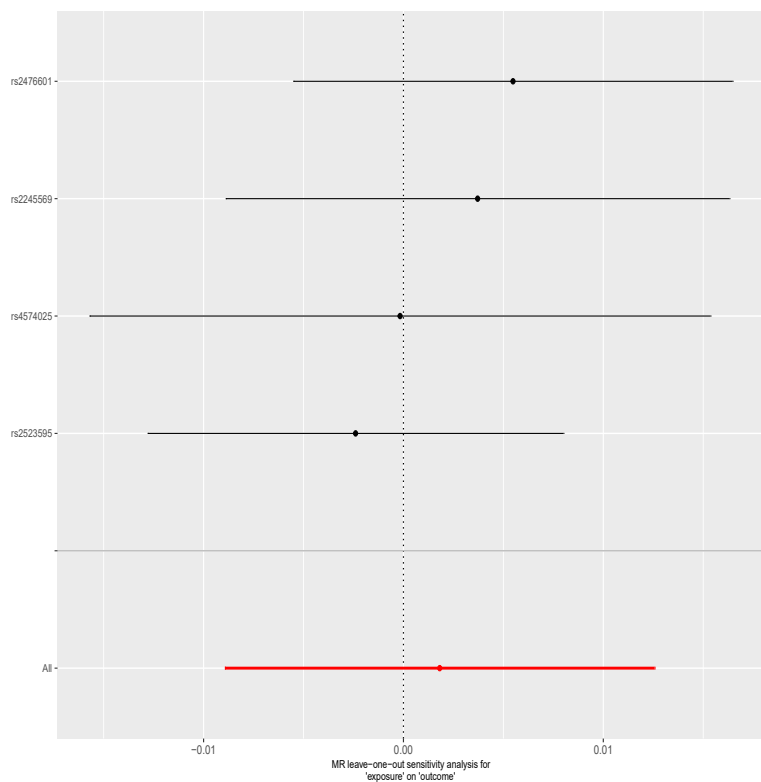

D

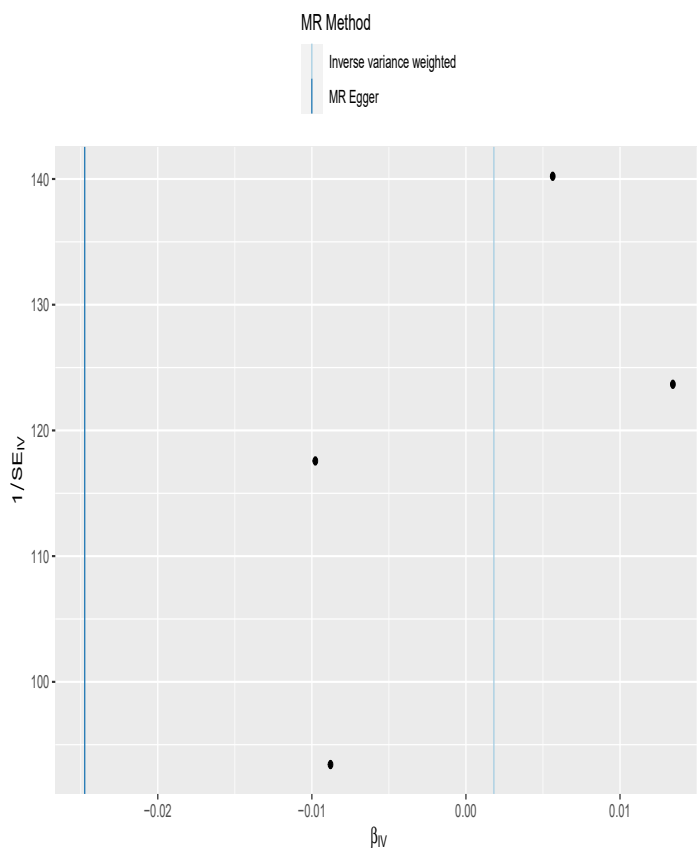

Supplement: Supplementary file 3 [file Image_2.PDF]

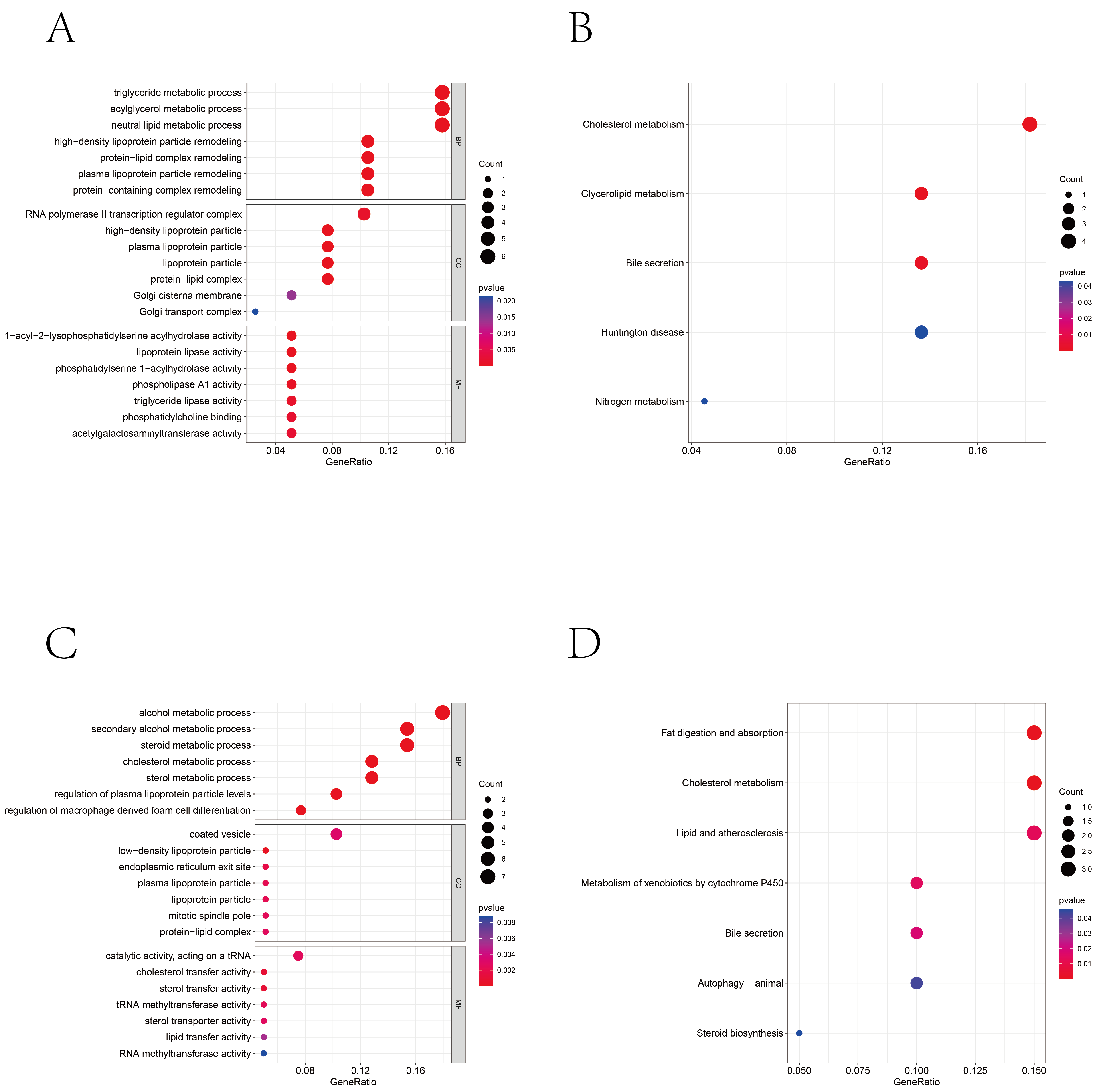

Supplement: Supplementary file 4 [file Image_3.TIF]
