# Supplementary material for: Causal effect of vitamin D on myasthenia gravis: a two-sample Mendelian randomization study
Source: Front Nutr. 2023 Jul 19;10:1171830. doi: 10.3389/fnut.2023.1171830 (PMC10394469; doi:10.3389/fnut.2023.1171830)
Supplement: Supplementary file 10 [file Data_Sheet_1.DOCX]

Supplementary Material

**Causal Effect of Vitamin D on Myasthenia Gravis: A Two-Sample Mendelian Randomization Study**

**Yidan Fan^1^, Xiashi Zeng^1^, Fenwei Lin^2^, Yuexuan Chen^1^, and Xu Chen^3^***

^1^The First Clinical Medical College, Guangzhou University of Chinese Medicine, Guangzhou, China

^2^Guangdong East Hospital of Guangmei Development Zone Hospital of the Third Affiliated Hospital of Sun Yat-sen University, Meizhou, China

^3^The First Affiliated Hospital of Guangzhou University of Chinese Medicine, Guangzhou, China

*** Correspondence:**

**Xu Chen**
61519764@qq.com

**Supplementary Table Legends**

Supplementary Table 1. Characteristics of SNPs in complete sets used in Mendelian Randomization analysis of effect of the serum 25(OH)D concentration on risk of myasthenia gravis.

Supplementary Table 2. Characteristics of SNPs in complete sets used in Mendelian randomization analysis of effect of risk of myasthenia gravis on the serum 25(OH)D concentration.

Supplementary Table 3. Characteristics of SNPs in outlier removed sets used in Mendelian randomization analysis of effect of risk of myasthenia gravis on the serum 25(OH)D concentration.

Supplementary Table 4. Gene region information corresponding to the instrumental variables obtained in the Phenoscanner database.

**Supplementary Figure Legends**

Supplementary Figure 1. Mendelian randomization study of the effects of serum 25(OH)D levels on risk of myasthenia gravis. (A) scatter plot, (B) forest plot, (C) leave-one-out sensitivity analysis, (D) funnel plot.

Supplementary Figure 2. Mendelian randomization study of the effects of risk of myasthenia gravis on serum 25(OH)D levels. (A) scatter plot, (B) forest plot, (C) leave-one-out sensitivity analysis, (D) funnel plot.

Supplementary Figure 3. (A) GO enrichment analysis of gene regions corresponding to SNPs associated with low vitamin D levels, (B) KEGG enrichment analysis of gene regions corresponding to SNPs associated with low vitamin D levels, (C) KEGG enrichment analysis of gene regions corresponding to SNPs associated with high vitamin D levels, (D) GO enrichment analysis of gene regions corresponding to SNPs associated with high vitamin D levels.

Supplementary Figure 4. Manhattan plot showing the correlation between SNPs and circulating vitamin D levels (where the green dots indicate the instrumental variables included in this study as circulating vitamin D levels, and the red line in the figure indicates that the threshold of *P* value is the genome-wide difference threshold 5 ×10^-8^)
